# Circulatory shear stress induces molecular changes and side population enrichment in primary tumor-derived lung cancer cells with higher metastatic potential

**DOI:** 10.1038/s41598-021-82634-1

**Published:** 2021-02-02

**Authors:** Keila Alvarado-Estrada, Lina Marenco-Hillembrand, Sushila Maharjan, Valerio Luca Mainardi, Yu Shrike Zhang, Natanael Zarco, Paula Schiapparelli, Hugo Guerrero-Cazares, Rachel Sarabia-Estrada, Alfredo Quinones-Hinojosa, Kaisorn L. Chaichana

**Affiliations:** 1 Department of Neurological Surgery, Mayo Clinic, 4500 San Pablo Road, Jacksonville, FL 32224 USA; 2 Division of Engineering in Medicine, Department of Medicine, Brigham and Women’s Hospital, Harvard Medical School, Cambridge, MA USA; 3 Regenerative Medicine Technologies Lab, Ente Ospedaliero Cantonale (EOC), Lugano, Switzerland; 4 Laboratory of Biological Structures Mechanics (LaBS), Department of Chemistry, Material and Chemical Engineering “Giulio Natta”, Politecnico Di Milano, Milan, Italy

**Keywords:** Cancer, Cancer microenvironment, Lung cancer, Metastasis

## Abstract

Cancer is a leading cause of death and disease worldwide. However, while the survival for patients with primary cancers is improving, the ability to prevent metastatic cancer has not. Once patients develop metastases, their prognosis is dismal. A critical step in metastasis is the transit of cancer cells in the circulatory system. In this hostile microenvironment, variations in pressure and flow can change cellular behavior. However, the effects that circulation has on cancer cells and the metastatic process remain unclear. To further understand this process, we engineered a closed-loop fluidic system to analyze molecular changes induced by variations in flow rate and pressure on primary tumor-derived lung adenocarcinoma cells. We found that cancer cells overexpress epithelial-to-mesenchymal transition markers TWIST1 and SNAI2, as well as stem-like marker CD44 (but not CD133, SOX2 and/or NANOG). Moreover, these cells display a fourfold increased percentage of side population cells and have an increased propensity for migration*. *In vivo*,* surviving circulatory cells lead to decreased survival in rodents. These results suggest that cancer cells that express a specific circulatory transition phenotype and are enriched in side population cells are able to survive prolonged circulatory stress and lead to increased metastatic disease and shorter survival.

## Introduction

**Figure 1:**
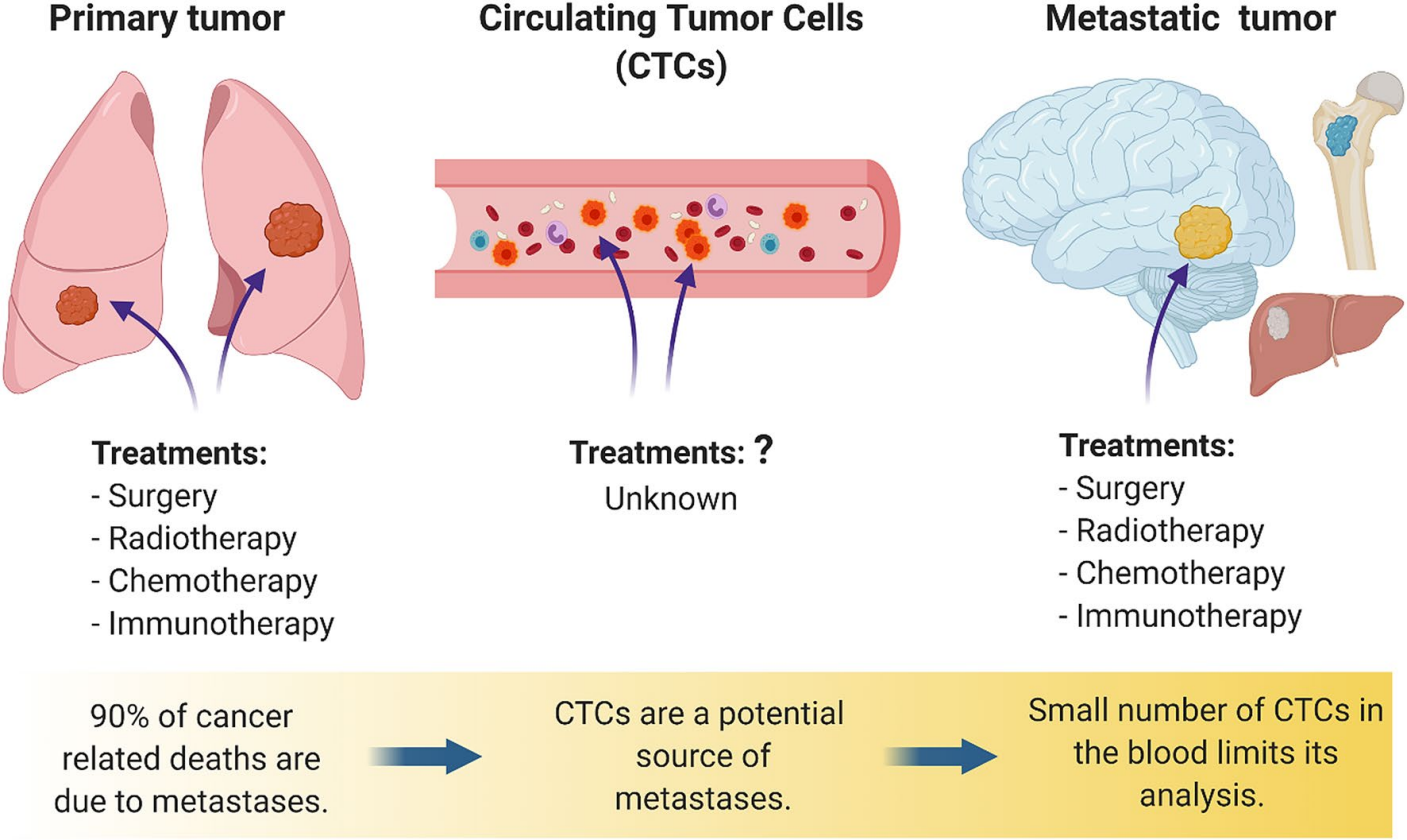
The cellular biology of cancer cells in circulation is poorly understood, hindering the development of efficient therapies. The majority of current treatments are aimed at treating the primary tumor or their metastatic deposits. There is little known about the treatment of cells in circulation, which are also called circulating tumor cells (CTCs). It remains unclear how some cells within the primary tumor can survive prolonged circulation to develop metastatic disease.

Cancer is the leading cause of death and disease in the U.S. with roughly 1.7 million new cancer cases each year^1,2^. While cancer costs the United States $173 billion annually, 70–80% of the costs occur in the treatment of metastatic cancer (MC). Once patients develop MC, their prognosis becomes exceedingly poorer^3^. Within the primary tumor, there is phenotypic and functional heterogeneity where different cancer cell populations can be found, each with specific characteristics and varying responses and ability to change and adapt to diverse microenvironments^4^. This cellular diversity and adaptability make treatments ineffective since a given treatment could kill some cells, while inducing adaptive resistance in others^5^. During metastasis, cells leave the primary tumor and migrate through tissues where they can enter circulation to later reach distant organs^6^. These circulating tumor cells (CTCs) can disseminate and therefore lead to poorer tumor control and poorer patient outcomes^7–9^. As with primary tumors, CTCs could also be heterogeneous and present phenotypic plasticity^10–13^. However, the phenotypic changes that some of these cells can undergo during circulation remain poorly understood (Fig. 1).

Several challenges hinder the possibility of characterizing these CTCs: (1) there is a low number of CTCs in the blood making them difficult to isolate^14^; (2) current technologies isolate CTCs based on their expression of specific markers, which could leave a significant portion of CTCs undetected^15–18^; and (3) most in vitro testing conditions do not recapitulate the circulatory microenvironment, where typical testing conditions place cells in suspension and not in the microenvironments seen in vivo^19,20^. Because of these limitations, it remains difficult to understand the molecular changes that cancer cells undergo when they reach and survive circulation thus making current therapies ineffective (Fig. 1). Therefore, in the present work, we analyze the effects of circulatory shear stress (CSS) on primary tumor-derived lung adenocarcinoma cells and hypothesize that a subset of these cells are able to survive prolonged CSS by adopting a unique molecular phenotype distinct from cancer cells within the primary tumor to establish the metastatic disease.

## Material and methods

### Cell culture

Human lung adenocarcinoma cell line A549 was obtained from American Type Culture Collection (ATCC, Manassas, VA, USA), and the cells were cultured in F-12 K medium (ATCC, Manassas, VA, USA) supplemented with 10% fetal bovine serum (Thermo Fisher Scientific, Waltham, MA, USA) and 1% penicillin/streptomycin/Amphotericin B (Thermo Fisher Scientific, Waltham, MA, USA). A549-green fluorescent protein (GFP)/firefly luciferase (*Luc)* cells stably expressing GFP and *Luc* genes were generated by adding the lentiviral particles directly to the culture medium with 4 µg/mL of polybrene (Sigma-Aldrich, St. Louis, MO, USA); a ratio of 5 lentiviral particles (LP)/cells was used for the transduction. After 48 h of incubation with lentivirus, the transduction medium was replaced with fresh complete medium to remove the virus and allow the cells to express the GFP and *Luc* reporter genes. The efficiency of transduced cells expressing GFP-*Luc* genes was calculated by confocal microscopy by detecting the GFP positive cells. Transduced cells were enriched by sorting [BD FACTSAria sorter III, Franklin Lakes, NJ, USA)] and cultured in fresh complete medium. Cells were seeded in suspension by culturing cells in 10-cm cell culture dishes that were coated with a thin layer of 1.2% agarose.

### Microfluidic system

**Figure 2:**
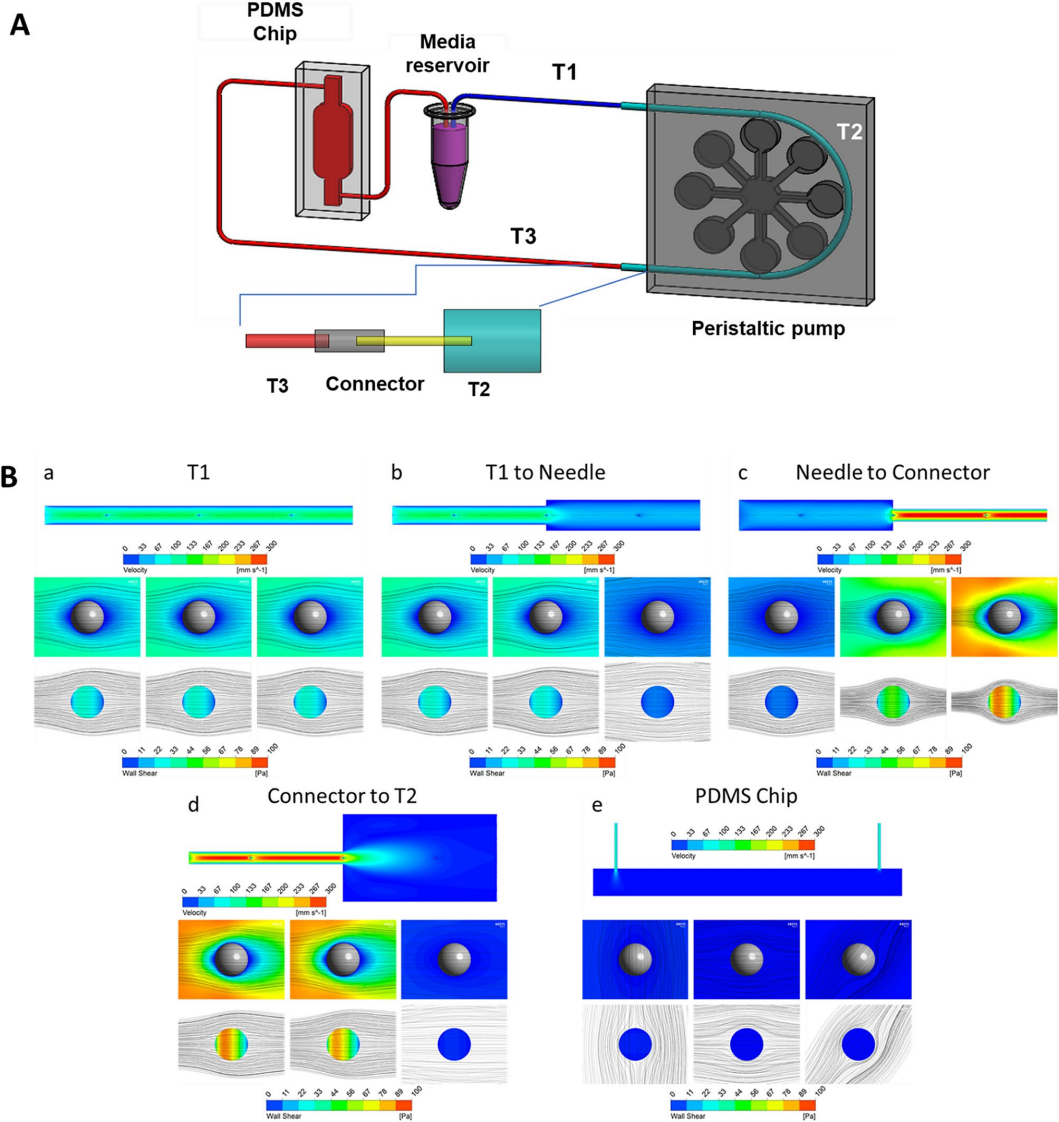
Variations in the internal diameter in the tubing system modifies the flow dynamics. Cancer cells can go from a static, attached state to being exposed to circulatory forces as they go from the primary tumor to their metastatic site. These different states can influence cancer cell viability and behavior. We designed a microfluidic system to recapitulate the circulatory system to simulate the drastic changes that occur in pressure and flow rate when cancer cells enter circulation. (**A)** Representative illustration of the microfluidic system and a segment of the tubing system with different internal diameters. (**B)** Simulation of flow dynamics changes due to changes in the tubing internal diameter. Flow streamlines and contours of circulatory shear stress on cancer cells that change in different segments of the system as measured by the Reynolds number (Re). (**a)** First tubing segment T1 (ID 0.304 mm) from the cell culture media reservoir; (**b)** Flow from T1 to T2 (ID 0.304 mm to ID 1.42 mm); (**c)** Flow from T2 to connector (ID 1.42 mm to ID 0.2 mm); (**d)** Flow from connector to T3 (ID 0.2 mm to ID 1.42 mm) (**e)** Flow through T3 (ID 0.30 4 mm); (**f**) Flow from T3 to the PDMS chip (ID 0.5–25mm^3^); (**g**) Flow through PDMS chip (25 mm^3^).

A circulating system with peristaltic flow was built in our laboratory (Fig. 2)^21,22^. The hydrodynamic parameters including the setting on the peristaltic pump [12.5 revolutions per minute (rpm)], the variable size of the tubing, and PDMS chip were done to best mimic what occurs in circulation in vivo. The tubing system with different internal diameters (IDs) and lengths and a polydimethylsiloxane (PDMS) chip were used to induce changes in the flow rate and pressure throughout the system, where the PDMS and its channels created a low flow and low stress site for cell visualization and viability assessments. The tubing was assembled as follows: two segments of tubing measuring 720 mm (0.304 mm ID, Microbore, Cole-Parmer, IL, USA) and 870 mm (0.304 mm ID) were connected to each side of a 410 mm tubing link (1.42 mm ID, Microbore) using home-made connectors (12 mm length) with a metallic cylinder (0.26 mm ID, stainless steel). They were inserted into a 25 mm length tubing (0.203 mm ID, Tygon), where one side of this tubing was connected to the medium reservoir (2 mL microtube) and the other side was connected to the PDMS chip [Inner chamber dimensions of 10 mm × 6 mm × 3 mm (l × b × h), with the channels on either side of the chamber measuring 8 mm each and a volume of 502.4 mm^3^]. The chip was then connected to the medium reservoir by a 200 mm length tubing (0.304 mm ID) to close the system. To generate peristaltic flow, a multichannel peristaltic pump (FH100M, Model 77724-02, Fisher Scientific, Pittsburgh, PA) was used (Supplementary Fig. S1). Cells were in constant circulation during 72 h in an incubator at 37 C and 5% CO_2_.

### Computational simulations

To analyze the fluid dynamics within the designed system and evaluate the effects on circulating cells, computational simulations were performed using a finite volume method (FVM) software (ANSYS Workbench, ANSYS Inc., Canonsburg, PA, USA). To reduce the global computational cost, the system was decomposed into its components and representative interfaces between coupled elements were selected for the simulations. For each simulation, three cells were considered as spherical solid bodies (Ø 20 µm) and located upstream, downstream and in correspondence of the interface setting a proper distance between two subsequent cells to avoid reciprocal influence. Symmetry boundary conditions were set on symmetrical planes parallel to the main direction of the flow, and a no-slip condition was set on system walls and on cell surfaces, constraining the fluid velocity to zero, while a zero-pressure condition was set on the system outlet.

The simulated volumes were divided into up to 3.7-M elements using ANSYS Meshing (ANSYS Inc., Canonsburg, PA, USA) adopting a tetrahedral grid scheme. Steady-state simulations were performed considering the culture medium as an aqueous solution with density and viscosity at 37 °C equal to 1006 kg/m^3^ and 1.13 × 10^–3^ Pa s, respectively. Inlet mass flow rate was defined in order to match the experimental condition of 300 µL/min, as set on the peristaltic pump. Fluid velocities in the tubes and flow-induced shear stresses on cell surface were extracted as parameters of interest. A dedicated simulation was then performed considering a single cell in the narrowest component of the system refining the mesh and increasing the number of elements up to 5 M to evaluate the recirculation regions immediately downstream of the cell. The parameters of the velocity of the peristaltic pump, tubing sizes and diameters were then altered to best match what occurs in vivo*.*

### Experimental groups

**Figure 3:**
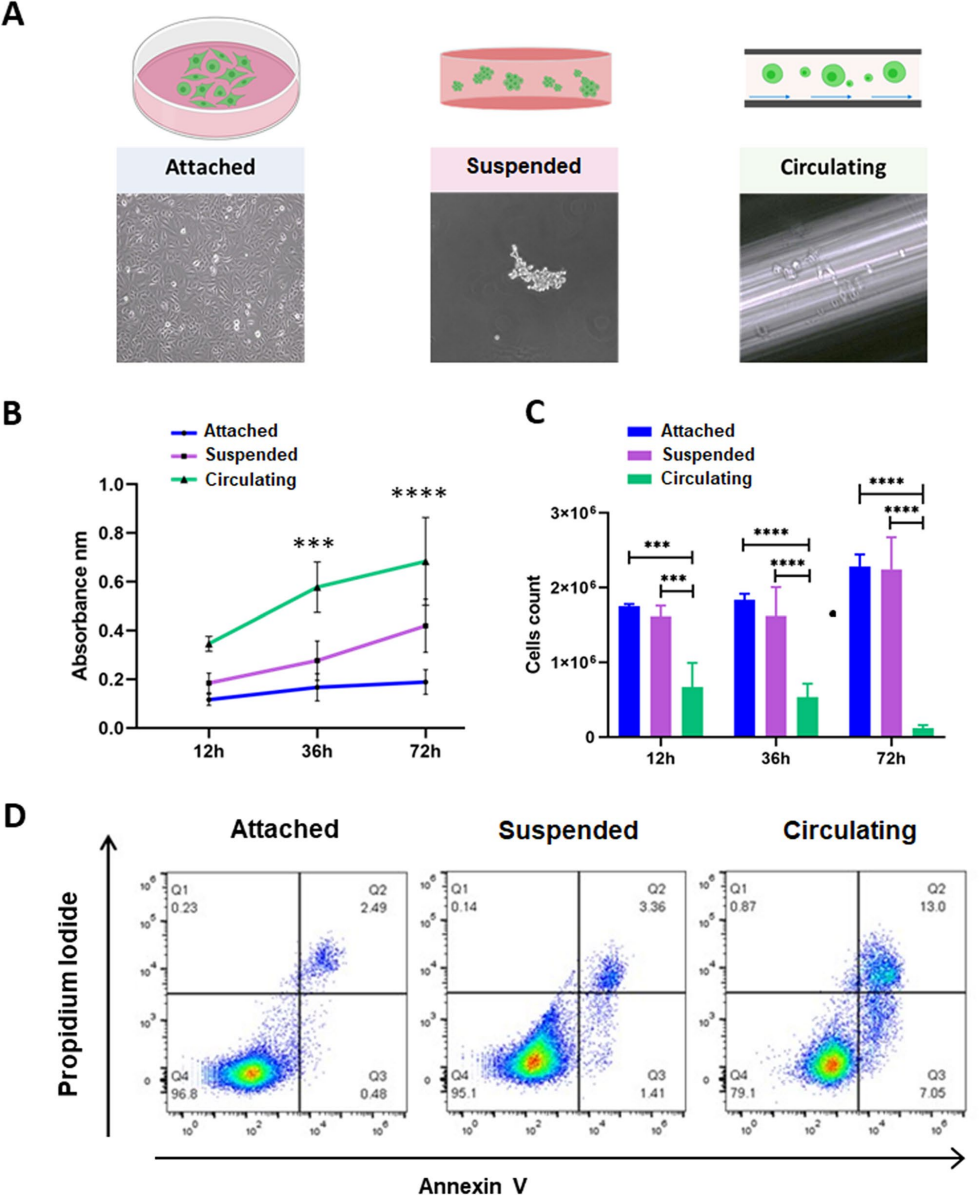
Circulation decreases primary lung cancer cell viability over time. Lung cancer cell viability was assessed using LDH and trypan blue for adhered, suspended, and circulatory primary lung cancer cells at different time points. Annexin V was performed to evaluate cell viability at 72 h. (**A**) Experimental conditions. Cells seeded in monolayer (attached), cells in suspension and cells in circulation. (**B**) LDH assay comparison over time. LDH assay showed that cellular damage increases significantly in cells subjected to circulation after 36 h, while cells in suspension experience significant cellular damage at 72 h as compared to attached cells 72 h (2-way ANOVA test, *p* < 0.005). (**C**) Cell counting comparison**.** Trypan blue assay showed that there was a significant decrease in cells in circulation starting at 12 h in circulation and most different at 72 h as compared to adhered and suspended cells (2-way ANOVA test, *p* < 0.0001). (**D**) Representative flow cytometry profiles and percentages of Annexin V+ cells, circulation increases apoptosis cell death during circulation in cancer cells after 72 h in circulation.; Q1: Necrosis, Q2: Late apoptosis, Q3: Early apoptosis, Q4: Live Cells.

Cells in circulation experience changes in flow dynamics and pressure and are in suspension while being dragged by the fluids. Mechanical conditions differ from one another and include static suspension, shear flow or 2D cell culture. We evaluated three different conditions: (1) static cells cultured in a bidimensional layer (adherent); (2) cells cultured in suspension (suspended) and (3) cells subjected to circulation (Fig. 3A). Each experimental group had a total of 2 × 10^6^ cells [5 × 10^5^ cells per microfluidic chip (n = 4) for circulating cells, 5 × 10^5^ cells per agarose plate (n = 4) for suspended cells, 5 × 10^5^ cells per petri dish (n = 4) for attached cells]. The group in suspension was used to differentiate cellular response to anoikis *versus* circulation. All experiments were done in triplicates.

### Determination of cellular damage

LDH release was performed on the supernatant collected at different time points (12, 36 and 72 h) for each group using the LDH-Cytox Assay Kit (BioLegend, San Diego, CA, USA). All samples were processed according to the manufacturer’s protocol. Briefly, 100 µL of supernatant from each condition was transferred to a 96-well plate, then 100 µL of working reagent was added to each well, the plate was incubated for 30 min at room temperature protected from light. The reaction was stopped by adding 50 µL of stop solution (1 M acetic acid) after incubation, and then absorbance was read at 490 nm using a plate-reading spectrophotometer (Synergy HTX Multi-Mode Microplate Reader, BioTek, Winooski, VT, USA). All assays were performed in triplicates.

### Gene expression profile

Total mRNA was extracted using the Trizol Reagent (Thermo Fisher Scientific, Waltham, MA, USA), and then purified using the RNeasy Mini Kit (Qiagen, Hilden, Germany) following the manufacturer’s protocol. RNA quantification was performed using NanoDrop One/One (Thermo Fisher Scientific, Waltham, MA, USA). Then, complementary DNA was synthesized using the SuperScript III Frist-Strand Synthesis System (Thermo Fisher Scientific, Grand Island, NY, USA). Quantitative RT-qPCR was performed using PowerUp SYBR Green Master Mix (Thermo Fisher Scientific,) and QuantStudio 3 Real Time PCR System (Applied Biosystems Foster City, CA, USA). The relative expression levels were calculated using the 2^−ΔΔCT^ method. The primer sequences used for qPCR are listed in Supplementary Table S1.

### Immunofluorescence

Cells subjected to suspension or circulation were washed with Hanks Balanced Salt Solution (HBSS) and centrifuged at 200* g* for 5 min at 4C. The supernatant was aspirated, and the cells were fixed in PFA 4% in PBS1x at room temperature for 15 min. After fixation, samples were centrifuged at 800* g* and washed with wash buffer (1× PBS/0.1% Triton × 100) three times for 5 min. After this, the cells were permeabilized for 15 min using PBS1× with 0.3% Triton X-100 and blocked using PBS1X/5% normal goat serum/0.3% Triton X-100 for 1 h at room temperature. After blocking, the samples were centrifuged at 800*g* for 5 min at 4C and resuspended in 100 uL of primary antibody mix (Supplementary Table S2) at 1:200 dilutions and incubated overnight at 4C. After overnight incubation, cells were centrifuged at 800*g* for 5 min at 4C, washed and resuspended in 100 uL of secondary antibody (Supplementary Table S3) at 1:500 dilution and incubated for 2 h at room temperature protected from light. After incubation, samples were washed and resuspended in mounting solution (10 uL of PVA-DABCO with DAPI). For attached cells, the cells were seeded in transparent coverslips (13 mm diameter, Thermanox, Nunc, Scientific Nunc Termanox Coverslips) into 24-well culture plates. After 72 h, cells were washed in PBS1× and fixed for 15 min in 4% formaldehyde in PBS. Attached cells were stained using the same conditions as stated above. Imaging was performed using a laser scanning confocal microscope, ZEISS LSM 800 (Carl Zeiss MicroImaging, Inc). Images were analyzed using the ZEN 2.3 (blue edition) software (Carl Zeiss, Jena, Germany).

### Apoptosis assay

Cells were harvested in cold phosphate-buffered saline (PBS). The samples were centrifuged at 300g for 5 min at 4 °C. Cells were resuspended in 1 × Annexin V binding buffer (Thermo Fischer Scientific), at a concentration of 1 × 10^6^ cells/mL. 100 µL of the main stock were distributed and stained with Alexa Fluor 488 Annexin V (1:20 dilution, Thermo Fisher Scientific) and propidium iodide (Thermo Fisher Scientific, V13241, 1:100 dilution). To assess metabolically active cells, the samples were additionally incubated with Calcein violet (1:50 dilution, Thermo Fisher Scientific). Samples were incubated for 30 min at room temperature in the dark. After incubation, cells were diluted with 400 µL of annexin 1 × binding buffer, mixed gently and kept on ice until the time of analysis. The samples were analyzed by flow cytometry using the Attune NxT flow cytometer (Thermo Fisher Scientific), and data were interpreted using FlowJo V10 software.

### Side population assay

Side population analysis was performed using the Vybrant Dye Cycle violet reagent (Thermo Fisher Scientific). Cells were sorted into side population and side population subgroups and were analyzed with flow cytometry and dye cycle violet assay. Percentage of side population cells was determined.

### Clonogenic assay

A fter 72 h of incubation, ~ 5 × 10^4^ cells from each condition (attached, suspended or circulating) were seeded in a 6-well plate and incubated for 12 h at 37 °C in 5% CO_2_. These cells were allowed to adhere for 12 h to provide sufficient time for the cells to attach and facilitate separating live from dead cells. These cells were then used to perform a 2D colony formation assay (CFA). Briefly, 50 cells from each condition were seeded into a 6-well plate and incubated for 10 days. The colonies were stained with 0.1% crystal violet (Millipore Sigma, St. Louis, MO, USA), each plate was imaged, and the number of colonies (> 500 µm) were quantified using the Fiji software (National Institutes of Health, 1.47v)^23^.

### Transwell migration assay

To determine cell migration, after 72 h of incubation, 2 × 10^6^ cells from each condition (attached, suspended, or circulating) were seeded in a 6-well plate and incubated for a period < 8 h at 37 °C in 5% CO_2_ (cells were allowed to adhere in order to facilitate separating live from dead cells). Cells were washed with PBS1X and detached using warmed Accutase. These cells were incubated in 6.5 mm in diameter, 8 µm pore transwell membranes, (Corning Incorporated, New York, NY, USA) at a cell density of 2 × 10^4^ cells suspended in 250 ul of serum-free F-12 K medium (ATCC 30-2004) per insert, in triplicates per each condition. The lower chamber was filled with 500 uL of F-12 K media containing 10% FBS. After 18 h the cells were fixed with 4% PFA. Non-migratory cells in the upper chamber were removed using a cotton swab. The membranes were dried and mounted using PVA DABCO with DAP at a 1:1000 dilution. Membranes were imaged and counted under the confocal microscope (nine random fields per membrane in triplicates). Cell migration was calculated as an average of migrating-cells per field.

### Animal experiments

All procedures were performed in accordance to relevant guidelines and regulations, adhering to ARRIVE guidelines, and approved by the Mayo Clinic Animal Care Institutional and Use Committee. 8-week female rats (athymic nude *Foxn1*^*RNU*^, Charles River) were injected in the left cardiac ventricle with 5 × 10^5^ A549 cells cultured under attached conditions or exposed to prolonged circulation (72 h) (previously transduced with lentiviral particles to constitutively express GFP-Luciferase gene) (n = 8 per group). Cells were resuspended in 250 µL of 1 × Dulbecco’s PBS (Thermo Fisher Scientific, Grand Island, NY, USA). Cell distribution and metastatic tumors were assessed and recorded weekly by bioluminescence imaging. In vivo bioluminescence images were acquired using the IVIS Spectrum In Vivo Imaging System (PerkinElmer, Waltham, MA, USA). Before imaging, d-luciferin (GoldBio, St. Louis, MO, USA) was injected intraperitoneally at a dose of 150 mg/kg and allowed to distribute for 10 min^24^. Rats were imaged once a week until sacrifice. Data acquisition and analysis were performed using the Living Image Software. Regions of interest were drawn, and the light emitted was recorded as the total flux (number of photons per second)^25^. To determine the number of metastases, region of interest (ROI) were traced on bioluminescence images, metastases were identified as ROIs were the total flux [p/s] was two times greater than the background. Animals were anesthetized using intraperitoneal ketamine (30–50 mg/kg; VETone Zetamine CIII, 100 mg/mL, MWI, Boise, ID) and xylazine (5–10 mg/kg; VETone XylaMed 100 mg/mL, MWI) and euthanized by intracardiac perfusion of 0.9% saline followed by 4% paraformaldehyde^26^. The organs were harvested and submitted for analysis. The tissues collected were embedded in paraffin and 5 µm sections were stained with hematoxylin and eosin for routine microscopic analysis.

### Statistics

All experiments were done in triplicates. Statistical analyses were performed using Graph-Pad Prism 8 software package (GraphPad Software, CA, USA). One-way ANOVA with Dunnett’s post hoc test was performed to compare multiple conditions against the control group, Tukey post hoc test to compare all pairs, or Student’s t-test to compare two conditions, whereby *= *p* < 0.05. For the survival experiment Graph-Pad Prism 8 software package was used to obtain the Kaplan–Meier survival and long-rank test.

## Results

### Microfluidic system that recapitulates the flow dynamics of the human circulatory system

The conditions that exist in circulation are different from other conditions, such as static suspension, shear flow, and adherence (Supplementary Figs. S2 and S3). Therefore, in order to simulate circulating conditions, we built a system with a PDMS chip and tubing of different diameters powered by a peristaltic pump and adjusted these parameters to make them more representative of what occurs in vivo (Fig. 2A, Supplementary Fig. S1). Computational analysis was performed to evaluate fluid dynamics effect on cancer cells in circulation, and, more specifically, the pressure drop (Δp) in kPA or mmHg, resistance (R) in Pa s/m^3^, mean fluid velocity V_m_ in mm/s, and Reynolds number (Re) for each component in the system (T1, connector needle, connector tube, T2, T3, and T4) (Supplementary Table S4). The effect of the fluid velocity on circulating cells was evaluated through computational simulations calculating the corresponding values of wall shear stress (WSS) on the cell surface in each of these components (T1, connector needle, connector tube, T2, T3, and T4) (Fig. 2, Supplementary Table S5). This device therefore has varying pressure changes, resistance, mean fluid velocity, Reynolds number, and wall shear stress that closely resemble to what occurs in vivo*,* as parts of the system closely resemble the magnitudes of WSS experienced in arterial (1–5.0 Pa) and venous (0.076–0.76 Pa) circulation^27–29^.

### Prolonged circulation of primary tumor-derived lung cancer cells selects for an exceedingly small subset of cells

To evaluate the viability of cells in prolonged circulation, we evaluated cell damage by measuring the amount of LDH secreted in the extracellular media of primary tumor-derived lung cancer cells under each different condition and at different times point (0, 12, 36 and 72 h) for each experimental group (attached, suspended, circulation, Fig. 3A). LDH levels were higher in the media from cells in circulation at every time point compared with cells cultured in attached or suspended conditions, where the highest difference started at 36 h and continued to 72 h in circulation (Fig. 3B, ANOVA *p* = 0.05). After 72 h under each condition, the cells were collected and counted by hemocytometer and trypan blue stain. From the group of cells subjected to circulation, only ~ 4% of viable cells were recovered after 72 h, where cells seeded in attached and suspension showed increased cell numbers due to proliferation (Fig. 3C).

Annexin V and propidium iodide were used to discriminate between early and late apoptosis. Annexin V is a protein with high affinity for phosphatidylserine, which is a compound that is translocated to the outer leaflet of cellular membrane of cells in early apoptosis^30,31^. Propidium iodide is a dye with affinity to DNA that is excluded from viable cells with intact membrane and permeable for dead and damaged cells in late apoptosis^32,33^. 2 × 10^6^ cells were injected into the system but only 3.6% was recovered after circulation. 7.05% of the surviving cells were in early apoptosis (Fig. 3D). After prolonged circulation, only a small subset of cancer cells survive whereby cell damage increases and more cells undergo apoptosis with increasing time.

### Primary tumor-derived lung cancer cells under CSS overexpress EMT makers but not stem-like markers

**Figure 4:**
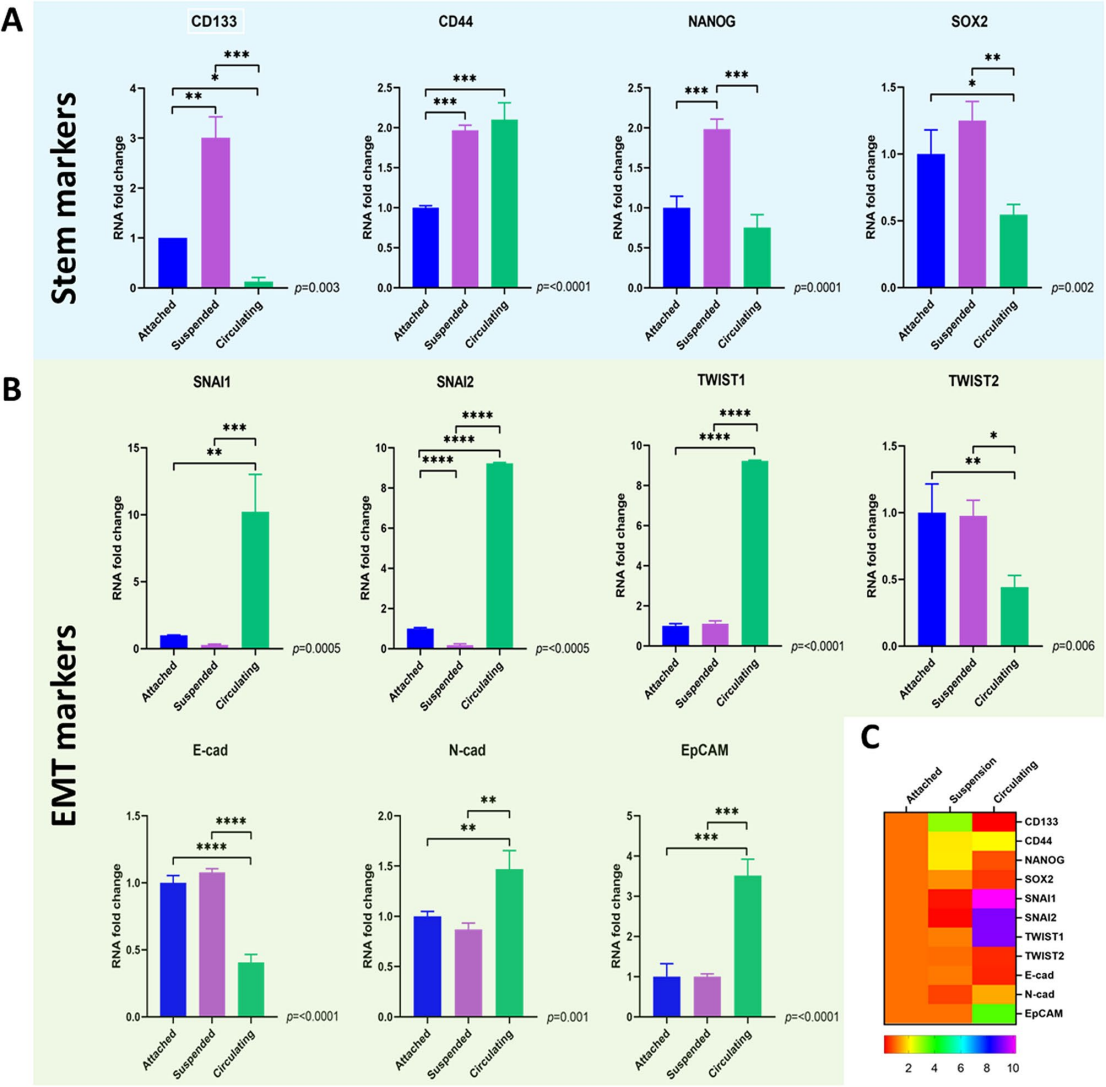
Lung cancer cells adopt a specific stem and EMT marker expression in response to circulation that is independent of a loss of adherence. The expression of stem makers and EMT expression in primary cancer cells in circulation, adherence, and suspension were assessed by qPCR. (**A**) Cells able to survive circulation downregulated stem markers CD133, NANOG and SOX2 but upregulated stem cell marker CD44 as compared to adherent cancer cells. Cells in suspension as compared to circulating cancer cells upregulated stem makers CD133, SOX2, NANOG but not CD44. (**B**) Cells able to survive circulation upregulate EMT markers N-Cad, EpCAM, SNAI1, SNAI2, and TWIST1, but down regulate TWIST2 and E-Cad as compared to attached cells and cells in suspension. SNAI2 was downregulated in cells in suspension as compared with attached and circulating cells. No significative differences were found in the expression of SNAI1, TWIST1, TWIST2, E-Cadherin, N-Cadherin and EpCAM as compared with attached cells. Thus, in response to circulation these primary lung cancer cells adopt a unique stem cell profile and EMT state to survive circulation that is independent of a loss of adherence. (**C**) Heatmap. Comparison of gene expression between experimental groups.

Only a small subset of cancer cells is able to survive circulation and generate metastatic tumors. It is unclear what cellular subtype is able to survive prolonged circulation, but many have hypothesized that these cells possess stem-like properties^34–36^ and/or undergo an epithelial-to-mesenchymal transition (EMT)^37,38^ in order to survive CSS and develop metastatic deposits^39–41^. To analyze the effect of CSS on the expressions of stem and EMT markers, qPCR was performed. After 72 h in circulation, significant differences were found in the expressions of stem-like and EMT markers between groups. For stem-like markers, cells in circulation had upregulation of CD44 (*p* ≤ 0.0001), but no significant differences were found in the expressions of stem-like markers CD133 (*p* = 0.003), NANOG (*p* = 0.0001) and SOX (*p* = 0.002) as compared to attached cells (Fig. 4A,C). For EMT markers, cells in circulation had upregulation of N-Cadherin (*p* = 0.001), SNAI1 (*p* = 0.0005), SNAI2 (*p* ≤ 0.0005) and TWIST1(*p* = 0.0001), and a down regulation of E-cadherin (*p* = 0.0001) and TWIST2 (*p* = 0.006), as compared to attached cells (Fig. 4B,C).

To differentiate the gene expressions due to lack of cell attachment, we compared circulating cells to suspended cells. For stem-like markers, cells in suspension overexpressed CD133 (*p* = 0.003), NANOG (*p* < 0.0001) and SOX2 (*p* = 0.002) as compared with attached cells and circulating cells, and no significant differences were found in the expression of CD44 (*p* > 0.05) as compared with circulating cells (Fig. 4A). For EMT markers, cells in suspension downregulated SNAI2 as compared with attached cells and circulating cells (Fig. 4B,C *p* = 0.0005).

**Figure 5:**
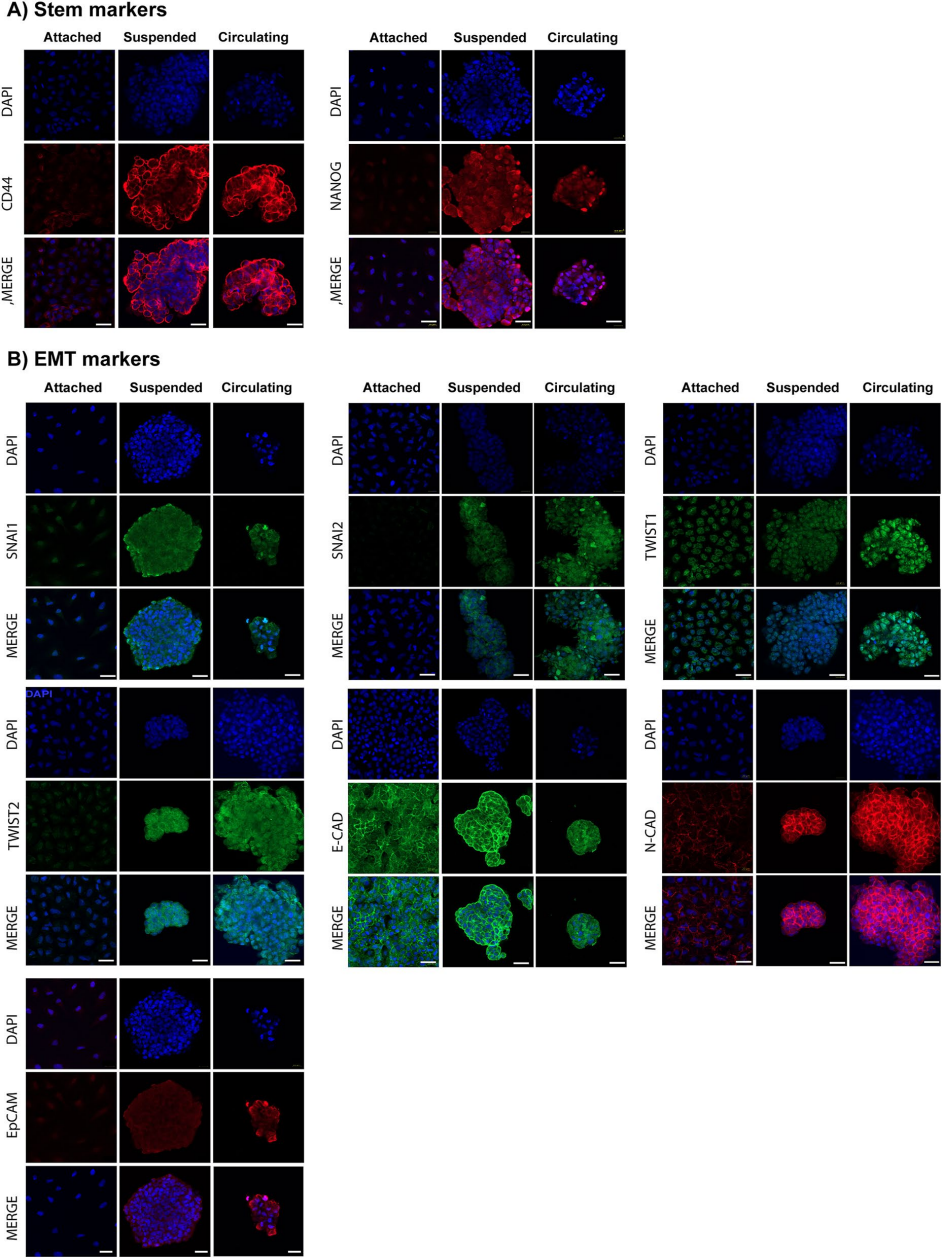
Immunofluorescence staining validating the results of stem and epithelial to mesenchymal transition (EMT)-related markers obtained from lung cancer cells after 72 h of adhesion, suspension or circulation. (**A**) Stem markers CD44 and NANOG, as well as nuclei, were primarily seen in circulating and suspended primary lung cancer cells, but not adherent lung cancer cells. (assessed with ZEN 2.3 Software, Carl Zeiss, Germany) (**B**) EMT markers SNAI1, SNAI2, TWST1, TWST2, E-Cadherin, N-Cadherin, Ep-CAM and nuclei revealed that cells subjected to circulation had increased expression of several EMT-related genes including (SNAI1, SNAI2, TWST1, TWST2) when compared to suspended and attached cells (assessed with ZEN 2.3 Software, Carl Zeiss, Germany). The adhesion marker, E-cadherin, was expressed in all conditions, but its immunoreactivity was more evident in cells subjected to suspension, followed by attached cells and least expressed in cells after circulation. N-cadherin was overexpressed in cells in circulation and suspension with an immunoreactivity that was more evident in the central region of the suspended and circulating cell spheroids and was expressed at very low levels in the adherent cells. Ep-CAM was differentially expressed through the three conditions with a marked increase in circulating cells when compared to suspended or adherent cells.

These results obtained by qPCR were validated using immunofluorescence. Overexpression of stem-like markers including CD44 and NANOG were observed in cells after circulation and suspension, with NANOG having an increased nuclear immunoreactivity in suspended vs. circulating cells. In contrast, these stem-like markers were almost undetectable in attached cells (Fig. 5A). Moreover, confocal microscopy revealed that cells subjected to circulation had increased expression of several EMT-related genes including (SNAI1, SNAI2, TWST1, TWST2) when compared to suspended and attached cells (Fig. 5B). In regard to adhesion molecules, E-cadherin was expressed in all conditions, but its immunoreactivity was more evident in cells subjected to suspension, followed by attached cells and least expressed in cells after circulation (Fig. 5B), N-cadherin was overexpressed in cells in circulation and suspension with an immunoreactivity that was more evident in the central region of the suspended and circulating cell spheroids, and was expressed at very low levels in the adherent cells (Fig. 5B). Notably, Ep-CAM was differentially expressed through the three conditions with a marked increase in circulating cells when compared to suspended or adherent cells (Fig. 5B).

Overall, these results suggest that circulating lung cancer cells represent a distinct population as compared to adherent cells whereby they express an EMT phenotype and selective stem cell markers, which is independent of loss of cell attachment that is seen in suspended cells.

### CSS enriches side population in primary tumor-derived lung cancer cells but does not affect the clonogenicity

**Figure 6:**
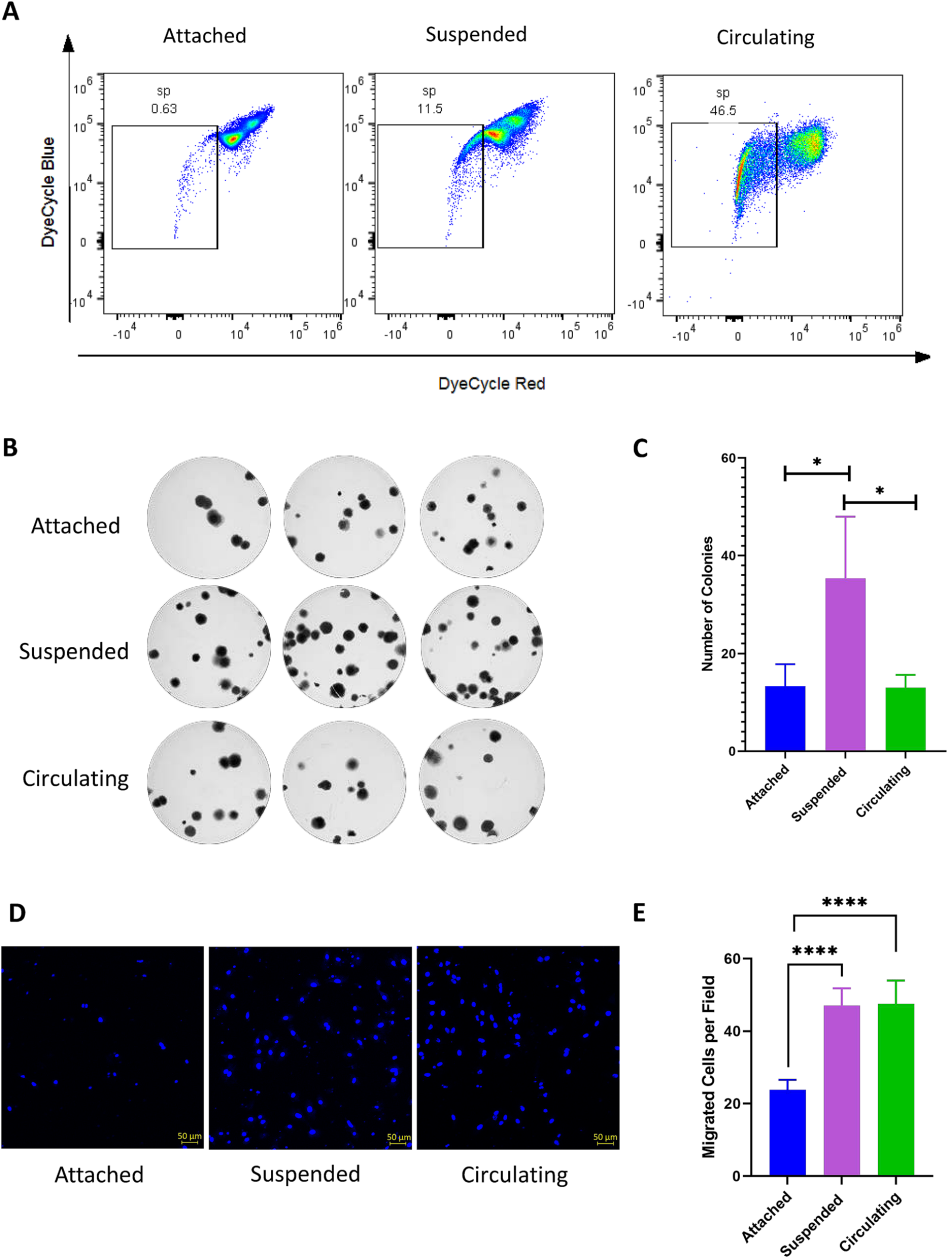
Circulation induces the enrichment of side population cells (SPCs) and increases lung cancer cell migration but does not alter cell clonogenicity. (**A**) Primary lung adenocarcinoma cells were stained with Vybrant DyeCycle Violet to determine dye effluxing side population cells. The percentage of SPCs was significantly higher in circulating cells as compared to attached cells and cells in suspension (**B**,**C**) Colony formation assay was performed to evaluate clonogenicity. The number of clonogenic colonies in circulating cells was not significantly different from attached cells but cells in suspension showed a higher number of colonies as compared with attached and circulating cells (*p* = 0.005). (**D**,**E**) Transwell migration assay showed that cells subjected to suspension and circulation presented higher invasive and migratory potentials than attached cells (*p* < 0.001). Representative microscopy image of DAPI staining for the 3 conditions (**D**) and statistical results of the mean ± SD (**E**). ****p < 0.001.

A cellular subpopulation that effluxes a specific DNA-binding dye is called side population (SP)^42,43^^.^ These cells have been shown to have stem-like phenotype, high tumorigenicity and drug resistance^44–46^. The effect of CSS on the enrichment of SP cells in primary tumor-derived lung cancer cells was analyzed by flow cytometry and Vybrant Dye Cycle Violet Stain, where fumitremorgin was used as transporter inhibitor. Lung cancers cells after 72 h in circulation showed a higher enrichment of SP as compared to cells in suspension and attached conditions (0.63%, 11.5% and 46% respectively) (Fig. 6A).

Colony formation assay also termed clonogenic assay, is an in vitro assay based on the ability of a single cell to grow into a colony through clonal expansion^47,48^. Clonogenic capacity has been proposed as an indicator of stemness^49^. To analyze the effect of CSS on the clonogenicity of cancer cells, CFAs were performed. No significant differences were found in the number of colonies between CSS and attached cells (Fig. 6B,C). These experiments show that circulation leads to enrichment of cells with side population characteristics that do not have clonogenicity, supporting that they are not stem like cells.

### CSS increases the migration of primary tumor-derived lung cancer cells

Cell migration and invasion are two essential processes involved in the seeding of cancer cells in distant organs^50^. It is known that mechanical cues can affect cell migration and that an EMT profile is related to increased cell motility and dissemination^51,52^. To examine the effects of CSS on primary cancer cell migration, transwell migration assays were performed. These primary cancer cells exposed to CSS or suspension for 72 h presented an increased number of cells migrated per field compared to attached cells (*p* < 0.001) (Fig. 6D,E). However, there were no statistically significant differences in migration in cells subjected to suspension versus circulation for a 72-h period (*p* = 0.2719) (Fig. 6E).

### Circulating primary tumor-derived cancer cells have increased in vivo metastatic capability

**Figure 7:**
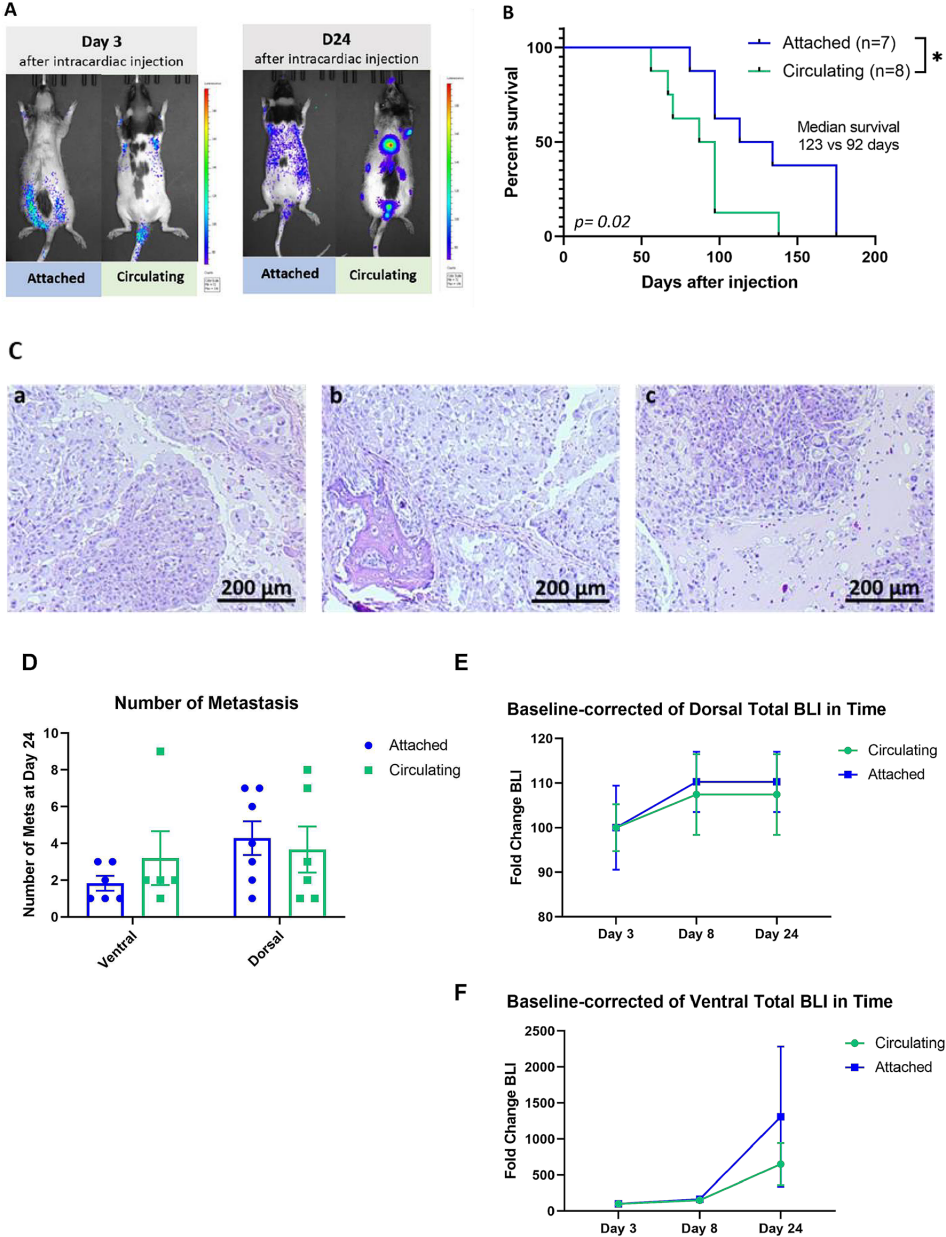
Lung cancer cells isolated from circulation lead to more metastatic disease and shorter survival than adherent cancer cells. 5 × 10^5^ cells seeded in attached cell culture conditions or subjected to circulation for 72 h were injected in the left ventricle of RNU rats, and the distributions of the cells and the metastasis were monitored through bioluminescence. (**A**) After 24 days post injection, the group injected with cells subjected to circulation developed higher number of metastatic deposits compared with the group of rats injected with cells under attached conditions. (**B**) Kaplan–Meier survival curve for rats with attached cells versus circulating cells. Rats injected with circulating cells survived on average 31 days less (attached cells n = 7; circulating cells n = 8) *p < 0.02 by long-rank test. (**C**) Hematoxylin and eosin staining of tissues with BLA signal. (**a–c**) Tissues from rats injected with circulating cells (jaw, spine, and knee) show formation of metastatic deposits in the jaw, spine, and knee. (**D**) Quantification of metastatic burden in rats as number of metastasis in rats intracardially injected with attached vs. circulating cells through bioluminescence imaging (BLI). (**E**) Baseline-corrected dorsal total BLI through time in attached versus circulating cells. (**F**) Baseline-corrected ventral total BLI through time in attached vs. circulating cells.

To evaluate the metastatic capability of cells under CSS, primary tumor-derived lung cancer cells subjected to circulation for 72 h and attached cells were injected intracardially in female athymic rats. Metastatic progression and distribution were monitored by bioluminescence imaging. When circulating primary tumor-derived lung cancer cells were injected, localized metastases appeared in 5 out of 8 rats at day 24 as compared to 2 out of 8 rats when attached primary tumor-derived lung cancer cells were injected (*p* = 0.05) (Fig. 7A,C). Moreover, rats injected with CSS cells survived on average 34 days less than those injected with attached cells (*p* = 0.02) (Fig. 7B). The quantification of metastatic burden was defined as number of metastasis detected through bioluminescence imaging (BLI). Our analysis did not show a statistically significant difference in the number of metastasis between rats injected with attached versus circulating cells in the ventral (*p* = 0.35) and/or dorsal (*p* = 0.69) views (Fig. 7D). There were also no statistical differences in the fold-change of BLI through time between the two conditions, in both ventral (*p* = 0.8, Day 24) and dorsal views (*p* = 0.5, Day 24) (Fig. 7E,F). Examples of tissues from rats injected with circulating cells showed formation of metastatic deposits in the jaw, spine, and knee (Fig. 6C,a-c respectively). This shows that circulating cancer cells more readily develop metastatic disease than adherent cancer cells, but not more metastatic burden, than adherent cells.

## Discussion

For decades, the study of metastasis has focused mainly on either the primary tumors or their metastatic deposits. However, little is known about cancer cells in circulation and if circulation selects for a specific cell type responsible for metastatic disease. When cells are in circulation, they are suspended in a liquid extracellular matrix and are exposed to constant changes in velocity and pressure as they are dragged through vessels of different sizes and structures with varying propulsive forces^53,54^. The mechanical and biological conditions associated with these circulatory stresses lead to cellular death in the vast majority of cells; however, it can also induce specific signals in some cells to allow them to survive and metastasize^55^. This adaptive response in certain cancer cells to these circulatory stresses is poorly understood. A better understanding of how these circulating cancer cells adapt and survive to circulation may lead to more effective strategies for preventing and treating metastatic disease, which is a major cause of morbidity and mortality for patients with a variety of primary cancers.

The ability to recapitulate what occurs in circulation is paramount to evaluating the effects that circulation and its associated forces have on primary cancer cells. As mentioned before, the physical conditions of cancer cells in circulation are specific but varied, where these cells are suspended while being dragged by a fluid with changes in flow rate and pressure. Most of the information regarding cells and flow had been obtained using parallel plate flow chambers, which is a system where cells are attached to a surface and the fluid passes over the cells to study their ability to resist the friction associated with flow^56–58^. Another system uses a hydrogel with embedded cancer cells with pulsatile fluid flow^59^. Cone and plate viscometers have also been used where cells are suspended but moving in circles in a plate^60,61^. Later systems used a syringe and tubing to generate flow over short distances^62,63^. Recently, similar systems with a continuous flow have been developed; however, most of the these contain a unique constriction and the circulatory time is around 24 h^64,65^. These models are limited because they do not truly study the cells in circulation for a prolonged period of time, but try and mimic the process whereby cells enter circulation or survive in circulation for a short duration. Our fluidic system was designed to induce non-attached cells to flow through tubing with different diameters to generate different exposures to velocity and pressure for a prolonged period of time. The hydrodynamic parameters including the setting on the peristaltic pump [12.5 Revolutions per minute, (rpm)], the variable size of the tubing, and PDMS chip were done to best mimic what occurs in circulation in vivo. The peristaltic pump was designed to replicate the pulsatility of the heart to generate fluid velocity whereby cells are squeezed through the pump, the size of the tubing to represent the shear forces that occur in the heart, arterial and venous vessels, and the PDMS chip to replicate the pressures within the capillary bed as it occurs in vivo^27,28,66^*.* Recent works have shown that cancer cells can survive longer periods of time in circulation in vivo and in vitro ^63,67,68^, and ours show that they can maintain viability in vitro for at least 72 h. In addition, to isolate the effects of CSS on CTCs, we also seeded cells in suspension and compared these cells to both attached and circulating cells. We also used cells from a primary tumor that had never been exposed to CSS^69^. In this way, we believe our model more accurately studies what happens to cells that can survive prolonged circulation similar to what has been proposed to occur in metastatic disease.

Previous studies have proposed that cancer cells that are more stem cell like and/or undergo EMT are better apt to survive in circulation to establish the metastatic disease^70,71^. Stem cells are thought to be able to withstand harsh environments and can recapitulate the primary tumor because of their multi-potential and clonogenic properties^72,73^. It has been shown that CTCs from patients over express CD133, CD44 and NANOG among other stem-like markers during early stages of breast cancer and this correlates with an invasive phenotype^74^. Furthermore, CD133 has been suggested as a marker for prognosis in several cancer types^75,76^. However the phenotype of cancer cells in circulation from patients is heterogeneous and could change according to the stage and type of cancer^77,78^. In this study, we found that CSS increase the percentage of SP cells, but they did not possess typical stem-like cell markers or increase in clonogenicity compared with cells non subjected to circulation. These cells expressed a unique phenotype where CD133, NANOG and SOX2 were downregulated and CD44 was significantly over expressed. In terms of functionality, these circulatory cells behaved more like SP cells without an increase in clonogenicity. In addition to stem-like cell characteristics, the majority of primary tumor cells are believed to possess an epithelial phenotype but undergo EMT to a mesenchymal phenotype in order to migrate through the extracellular matrix^37,38,79,80^. However, once these cancer cells enter the circulation, it is unclear what phenotype they adopt. Some studies report these cells are mainly epithelial while other studies suggest that these cells are mesenchymal in phenotype and others argue these cells adopt a hybrid phenotype where mesenchymal and epithelial markers are co-expressed^81–86^. In this study, we found that SNAI2 and TWIST1 were upregulated in cells able to survive circulation. These cells were not entirely epithelial and not entirely mesenchymal. In order to better isolate the effects of circulatory stresses on the cells, we wanted to eliminate the effects of loss of cell attachment and anoikis on stem cell and EMT markers as well as side population enrichment. Therefore, we compared cells in suspension, presumably as an intermediate state between attachment and circulation. We found that suspended cells due to their loss of adhesion, behaved more similarly with stem cells displaying higher proliferative ability and upregulation of stem cell markers CD133, NANOG, and SOX2. Cells that develop metastatic disease therefore adopt a unique phenotype to survive circulation and are distinct from the primary tumor. In the in vivo experiments, we found that animals injected with circulating cancer cells more frequently developed metastatic disease but not more disease burden. However, animals injected with circulating cancer cells, despite having similar disease burdens, had decreased overall survival.

### Limitations

This study was designed to evaluate the effects of circulatory stresses on primary cancer cells. The in vitro conditions were carried out with a microfluidic device to mimic what occurs in vivo by having a peristaltic pump, tubing, PDMS chip, and tubing to replicate the heart, arteries/arterioles, capillaries, and veins, respectively. We attempted to isolate the effects of circulation on the cells in vitro by removing cell attachment as well as comparing circulating cells to not only attached cells, but also cells in suspension. We also evaluated the role of invasion, side population enrichment, and clonogenicity after cells were exposed to circulatory stress. This is the first study of its kind to evaluate these effects on primary lung cancer cells. There are some limitations, however. The peristaltic pump, while functioning to power the microfluidic system, relies on tubing to be squeezed and therefore the cells will be squeezed through this device adding additional shear forces that may not accurately represent the heart. Although knowing the level of shear stress on circulating cells in the peristaltic pump would be very interesting and would add precious information, it is also true that this type of analysis requires a dedicated study with more complicated and time-dependent simulations that are very costly from a computational point of view. Moreover, peristaltic pumps are generally designed in a way in which the tube is not completely occluded by the rollers. This aspect has the double advantage of not damaging the tube itself during use while decreasing the stress applied to circulating material. For this reason, circulating cells should not undergo a detrimental level of shear stress when passing through the pump system. In addition, the effect of shear stress on cell viability is due to a combination of level of stress and time of exposure. Considering the length of the whole circulating system, we hypothesize that the time spent by cells in proximity of the pump rollers is short enough to not represent the most detrimental component of the circuit. However, further studies would help in this understanding. In the in vivo experiments, we only compared adhered cells to circulatory cells in regard to their metastatic potential and effect on survival. We limited the experiment to these two cohorts in order to compare the primary tumor (adherent cells) with circulatory cells. We did not add the suspended cells under the assumption that adherent cells that suspend represent an intermediate state between the primary tumor and circulatory cells, and therefore just wanted to compare adherent with circulatory cells. In addition, we were limited in terms of resources to not only add a third experimental group that may confound the data, but also in terms of the number of animals used for the experimentation. Further experiments expanding the number of animals and using suspended cells may provide additional insight into the effects of circulatory stresses on in vivo metastatic potential.

## Conclusions

In summary, this study found a cellular subpopulation of cancer cells from primary tumors that are able to survive in circulation for several days and have a high metastatic capacity. Surviving cells showed a specific phenotype that includes a high percentage of side population cells and overexpression of CD44. These findings contribute to understanding the cellular response to a circulatory microenvironment.

## Supplementary Information

Supplementary Information
