# Supplementary material for: Circulatory shear stress induces molecular changes and side population enrichment in primary tumor-derived lung cancer cells with higher metastatic potential
Source: Sci Rep. 2021 Feb 2;11:2800. doi: 10.1038/s41598-021-82634-1 (PMC7854722; doi:10.1038/s41598-021-82634-1)
Supplement: Supplementary file 1 — Supplementary Information [file 41598_2021_82634_MOESM1_ESM.docx]

Supplementary Material

Circulatory Shear Stress Induces Molecular Changes and Side Population Enrichment in Primary Tumor-derived Lung Cancer Cells with Higher Metastatic Potential

Keila Alvarado-Estrada,^1^ Lina Marenco-Hillembrand,^1^ Sushila Maharjan,^2^ Valerio Luca Mainardi,^2-4^ Yu Shrike Zhang,^2^ Natanael Zarco,^1^ Paula Schiapparelli,^1^ Hugo Guerrero-Cazares,^1^ Rachel Sarabia-Estrada,^1^ Alfredo Quinones-Hinojosa,^1^ Kaisorn L. Chaichana^1*^

^1^Department of Neurological Surgery, Mayo Clinic, FL, USA

^2^Division of Engineering in Medicine, Department of Medicine, Brigham and Women’s Hospital, Harvard Medical School, Cambridge, MA, USA

^3^ Regenerative Medicine Technologies Lab, Ente Ospedaliero Cantonale (EOC), Lugano, Switzerland

^4^ Laboratory of Biological Structures Mechanics (LaBS), Department of Chemistry, Material and Chemical Engineering “Giulio Natta”, Politecnico di Milano, Milan, Italy

Supplementary Figure 1. Microfluidic system dimensions and assembly


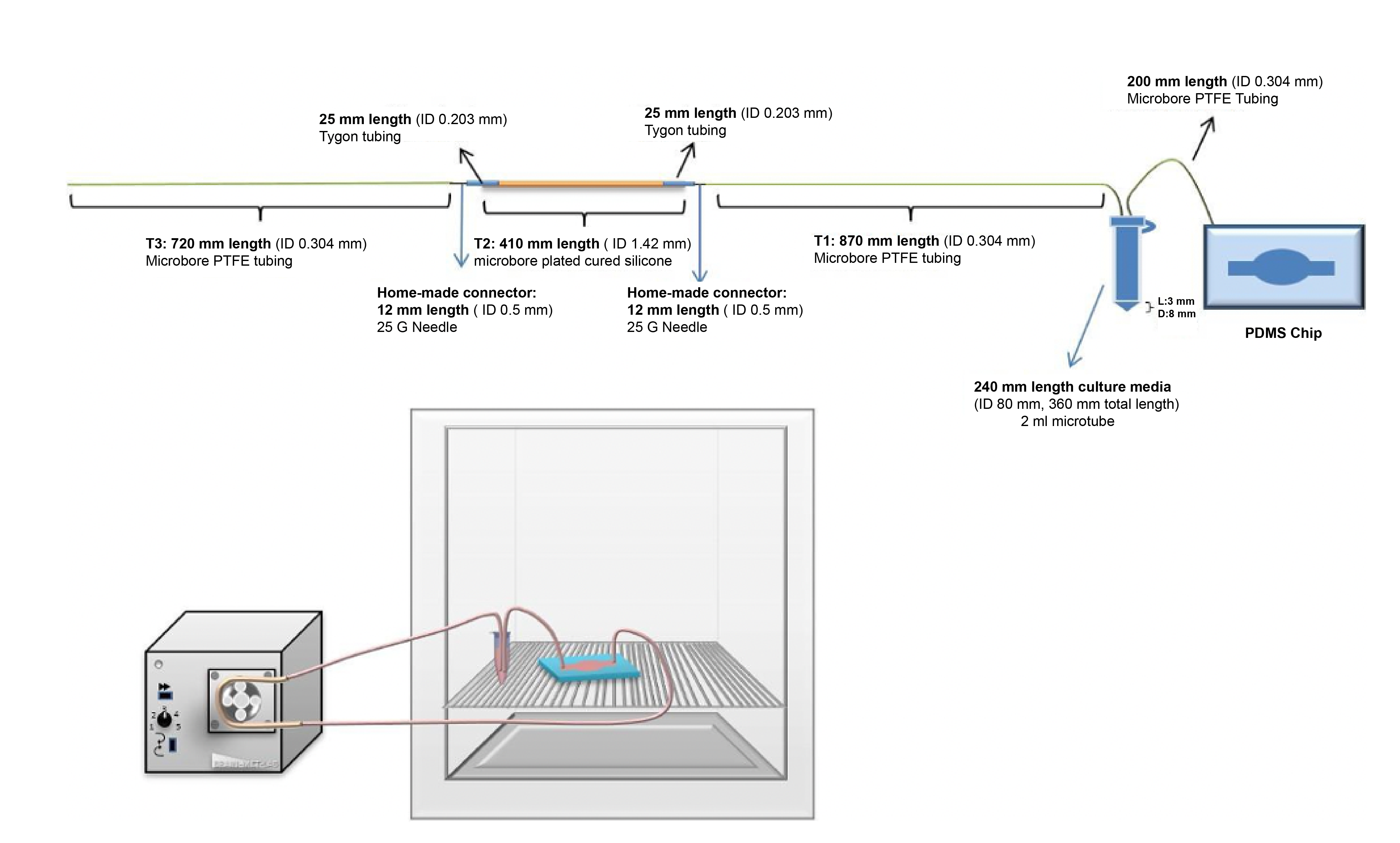


Supplementary Figure 2. Stem maker expression on cells subjected to 2D cell culture, shear flow and circulation


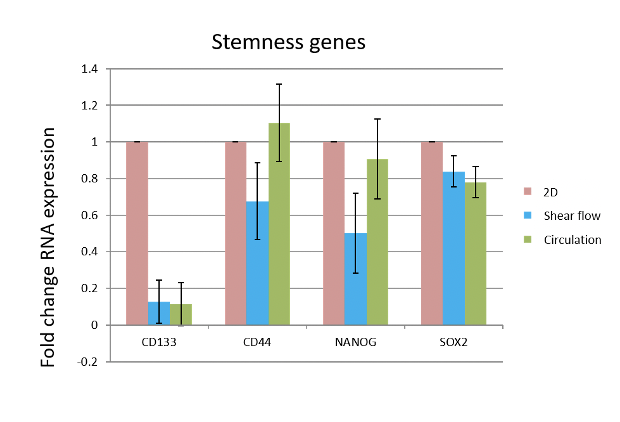


Supplementary Figure 3. EMT marker expression on cells subjected to 2D cell culture, shear flow and circulation


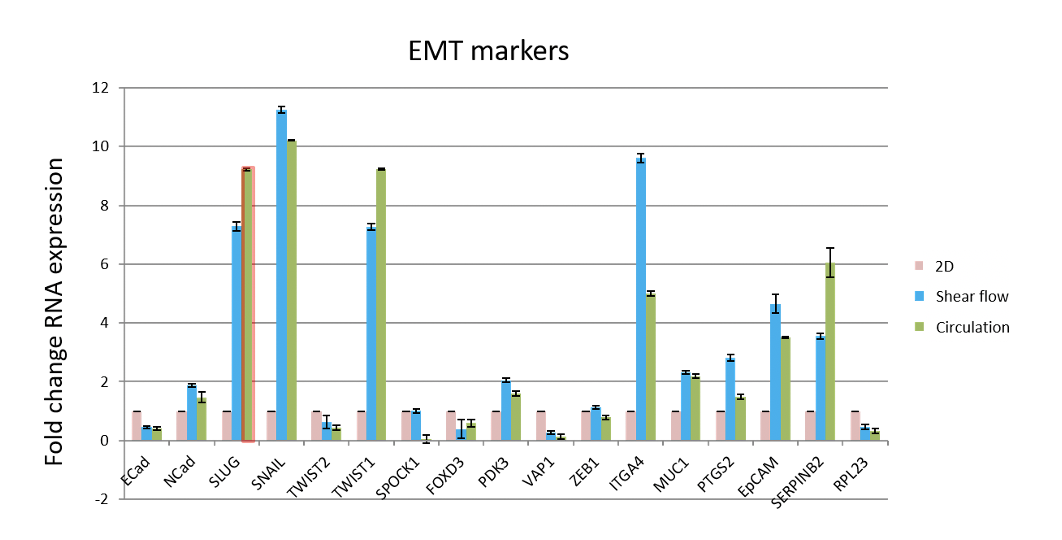


Supplementary Table S1. Primer Sequence Obtained from PrimerBank- MGH-PGA

| NANOG | TTTGTGGGCCTGAAGAAAACT | AGGGCTGTCCTGAATAAGCAG |
| --- | --- | --- |
| SOX2 | CTCGTGCAGTTCTACTCGTCG | AGCTCTCGGTCAGGTCCTTT |
| CD133 | GGCCCAGTACAACACTACCAA | ATTCCGCCTCCTAGCACTGAA |
| CD44 | CTGCCGCTTTGCAGGTGTA | CATTGTGGGCAAGGTGCTATT |
| SNAI1 | TCGGAAGCCTAACTACAGCGA | AGATGAGCATTGGCAGCGAG |
| SNAI2 | CGAACTGGACACACATACAGTG | CTGAGGATCTCTGGTTGTGGT |
| TWIST1 | GCCTAGAGTTGCCGACTTATG | TGCGTTTCCTGTTAAGGTAGC |
| TWIST2 | AGCAAGAAGTCGAGCGAA | CAGCTT GAGCGTCTGGATCT |
| E-Cadherin | CCTAGATGAACCTTATGAGGCCA | GCTGTAGAGGAGACGAGCATTAT |
| N-Cadherin | TGTATGTGGGCAAGATCCACT | CTCGTCGATCAGGAAGATGGT |
| EpCAM | TGATCCTGACTGCGATGAGAG | CTTGTCTGTTCTTCTGACCCC |
| SPOCK1 | CCCAACCACGGCAATTTCCTA | ATCGTCTCGAAAGCGGTTCC |
| FOXD3 | GACGACGGGCTGGAAGAGAA | GCCTCCTTGGGCAATGTCA |
| PDK3 | CGCTCTCCATCAAACAATTCCT | CCACTGAAGGGCGGTTAAGTA |
| VAP1 | ACCTGGTTTGGCCTCTACTAC | GGCCTTGTGGTTCACTAGCA |
| ZEB1 | TTACACCTTTGCATACAGAACCC | TTTACGATTACACCCAGACTGC |
| ITGA4 | TACAGATGCAGGATCGGAAAGA | TACAGATGCAGGATCGGAAAGA |
| MUC12 | CCAGTTCAAGCGACCCTTTTA | CGCTGTGGGATACTGTTGATT |
| PTGS2 | TAAGTGCGATTGTACCCGGAC | TTTGTAGCCATAGTCAGCATTGT |
| SERPINB2 | AAATGGGCTTTATCCTTTCCGT | AGCTTTTCACGCAAGTACATCA |
| RPL23 | TCCTCTGGTGCGAAATTCCG | CGTCCCTTGATCCCCTTCAC |
| GAPDH | CTCCTCCTGTTCGACAGTCAGC | CCCAATACGACCAAATCCGTT |

Supplementary Table S2. Primary Antibodies for Immunofluorescence

| **Target** | **Catalog Number** | **Host** | **Isotype** | **Dilution** | **Company** |
| --- | --- | --- | --- | --- | --- |
| CD44 | 14-0441-82 | Rat | IgG2b/Kappa | 1:200 | Invitrogen |
| SOX2 | sc-17320 | Goat | Goat IgG | 1:200 | Santa Cruz  Biotechnology |
| NANOG | 4893 | Mouse | Mouse IgG1 | 1:200 | Cell Signalling |
| SNAIL | MA5-14801 | Rabbit | Rabbit IgG | 1:200 | Invitrogen |
| SLUG | 9585 | Rabbit | Rabbit IgG | 1:200 | Cell Signalling |
| TWIST1 | ABD29 | Rabbit | Rabbit IgG | 1:200 | Millipore Sigma |
| TWIST2 | SAB1401972-  100UG | Rabbit | Rabbit IgG | 1:200 | Millipore Sigma |
| E-Cadherin | 13116 | Rabbit | Rabbit IgG | 1:200 | Cell Signalling |
| N-Cadherin | 14215 | Mouse | Mouse IgG1 | 1:200 | Cell Signalling |
| Ep-CAM | 2929 | Mouse | Mouse IgG1 | 1:200 | Cell Signalling |

Supplementary Table S3. Secondary Antibodies for Immunofluorescence

| **Fluorophore** | **Catalog Number** | **Host** | **Isotype** | **Dilution** | **Company** |
| --- | --- | --- | --- | --- | --- |
| 488 | A-27034 | Goat | Rabbit IgG | 1:500 | Invitrogen |
| 647 | A-21235 | Goat | Mouse IgG | 1:500 | Invitrogen |
| 647 | A-21247 | Goat | Rat IgG | 1:500 | Invitrogen |
| 647 | # PA1-28738 | Donkey | Goat IgG | 1:500 | Invitrogen |

Supplementary Table S4. Hemodynamic parameters within each component of the microfluidic system

|  | **Diameter** | **Length** | **Pressure drop** | **Pressure drop** | **Resistance** | **Velocity** | **Reynolds** |
| --- | --- | --- | --- | --- | --- | --- | --- |
|  | ID [mm] | l [mm] | Δp [kPa] | Δp [mmHg] | R [Pas/m^3^] | vm [mm/s] | Re |
| Tube 1 | 0.304 | 720 | 19.79 | 148.47 | 3.88E+12 | 70.26 | 19 |
| Needle ID 0.5 | 0.500 | 12 | 0.05 | 0.34 | 8.84E+09 | 25.97 | 12 |
| Connector | 0.203 | 25 | 3.46 | 25.93 | 6.78E+11 | 157.58 | 28 |
| Tube 2 (pump) | 1.420 | 410 | 0.02 | 0.18 | 4.64E+09 | 3.22 | 4 |
| Connector | 0.203 | 25 | 3.46 | 25.93 | 6.78E+11 | 157.58 | 28 |
| Needle ID 0.5 | 0.500 | 12 | 0.05 | 0.34 | 8.84E+09 | 25.97 | 12 |
| Tube 3 | 0.304 | 870 | 23.92 | 179.40 | 4.69E+12 | 70.26 | 19 |
| Eppendorf | NA | NA | NA | NA | NA | NA | NA |
| Tube 4 | 0.304 | 200 | 5.50 | 41.24 | 1.08E+12 | 70.26 | 19 |
| Chip | NA | NA | NA | NA | NA | NA | NA |

**Supplementary Table S5. Wall shear stresses within each component of the microfluidic system**

|  | **Diameter** | **Length** | **Mean wall shear stress** | **Max wall shear stress** |
| --- | --- | --- | --- | --- |
|  | ID [mm] | l [mm] | WSS [Pa] | WSS [Pa] |
| Tube 1 | 0.304 | 720 | 23.88 | 33.24 |
| Needle ID 0.5 | 0.500 | 12 | 7.79 | 10.36 |
| Connector | 0.203 | 25 | 62.12 | 91.88 |
| Tube 2 (pump) | 1.420 | 410 | 2.60 | 3.48 |
| Connector | 0.203 | 25 | 62.12 | 91.88 |
| Needle ID 0.5 | 0.500 | 12 | 7.79 | 10.36 |
| Tube 3 | 0.304 | 870 | 23.88 | 33.24 |
| Eppendorf | NA | NA | NA | NA |
| Tube 4 | 0.304 | 200 | 23.88 | 33.24 |
| Chip | NA | NA | 0.05 | 0.06 |
